# Clonorchiasis control: starting from awareness

**DOI:** 10.1186/2049-9957-3-33

**Published:** 2014-09-15

**Authors:** Men-Bao Qian

**Affiliations:** 1 National Institute of Parasitic Diseases, Chinese Center for Disease Control and Prevention; WHO Collaborative Center for Malaria, Schistosomiasis and Filariasis; Key Laboratory of Parasite and Vector Biology, Ministry of Health, Shanghai, China

**Keywords:** *Clonorchis sinensis*, Clonorchiasis, Cholangiocarcinoma, Health education, Television

## Abstract

Clonorchiasis is caused by infection with food-borne liver fluke, namely *Clonorchis sinensis*, which is also considered to be a neglected tropical disease. It is estimated that over 10 million people are infected with *C. sinensis* in China and, subsequently, several thousand new cholangiocarcinoma cases occur annually. On May 18, 2014, China Central Television broadcasted an episode on the habit of raw-fish eating and its potential to cause clonorchiasis in a programme called *Health on the tip of the tongue*. Here, I briefly introduce the content of the episode and discuss its significance for clonorchiasis control in China.

## Multilingual abstracts

Please see Additional file 1 for translations of abstract into the six official working languages of the United Nations.

Clonorchiasis is caused by infection with the liver fluke *Clonorchis sinensis,* which is considered to be a neglected tropical disease [1]. Chronic *C. sinensis* infection is associated with liver and biliary conditions, such as gallstone, cholecystitis, cholangitis and cholangiocarcinoma (CCA) [1,2]. It is estimated that over 10 million people are currently infected with *C. sinensis* in China and several thousand new CCA cases related to this infection occur annually [3,4].

Unawareness and neglect are the main reasons why prevalence of infection and the public health burden of clonorchiasis in China are so high. Yet, it is conceivable that this will change in the future. On May 18, 2014, China Central Television broadcasted an episode on the habit of raw-fish eating and its potential to cause clonorchiasis in a programme called *Health on the tip of the tongue* (http://news.cntv.cn/2014/05/18/VIDE1400389298866439.shtml). In this 12-minute-long episode, the prevailing culture of the habit of raw-fish eating in southeastern and northeastern China was firstly introduced. After this, a patient with CCA caused by the *C. sinensis* infection was interviewed in order to elucidate the impairment of clonorchiasis due to raw-fish eating. The episode went on to show that restaurants which served raw-fish dishes believed that their fish was safe and without *C. sinensis* infection because the fish was caught in large rivers. This was refuted after the fish was examined. Many of the inhabitants interviewed said that they knew of clonorchiasis and also believed that *C. sinensis* could be killed by various condiments with the meals, such as mustard, garlic, vinegar and, especially, wine. These condiments were tested in an experiment, but no evidence to support these views presented itself. Finally, key messages, such as avoiding consumption of raw fish, and getting examined and treated in clinics after eating raw fish, were expressed.

As the most influential media outlet in China, the fact that China Central Television broadcasted an episode disseminating information on the transmission and prevention of clonorchiasis demonstrates the media’s awareness of the disease burden caused by clonorchiasis. It is also instrumental in providing wide-scale health education for Chinese, particularly to that population that has the habit of eating raw fish. As a large population is infected with *C. sinensis* chronically which can consequently cause CCA in the long term, a lot of effort is still required to tackle clonorchiasis in China [4]. But the participation of the main media such as China Central Television is positive and encourages the belief that clonorchiasis can be eliminated in the future. However, more local TV in clonorchiasis endemic areas is needed to strengthen this health education campaign to promote the control of clonorchiasis in China.

## Competing interests

The author declares that he has no competing interests.

## Supplementary Material

Additional file 1 Translation of the abstract into the six official working languages of the United Nations. Click here for file
