# Supplementary material for: Clonorchiasis control: starting from awareness
Source: Infect Dis Poverty. 2014 Sep 15;3:33. doi: 10.1186/2049-9957-3-33 (PMC4168994; doi:10.1186/2049-9957-3-33)
Supplement: Additional file 1 — Translation of the abstract into the six official working languages of the United Nations. [file 2049-9957-3-33-S1.pdf]

Translation of the abstract into the six official working languages of the United Nations

السيطرة على داء متفرعات الخصية يبدأ بالوعي

مين-باو تشيان

المستخلص :

يحدث داء متفرعات الخصية نتيجة العدوى بدودة كبدية تنقله عبر الأغذية، وخاصة *Clonorchis sinensis*، والذي يعتبر أيضا أحد أمراض المناطق المدارية المهملة. ويقدر أن أكثر من 10 ملايين شخص مصابين بفيروس *C. sinensis* في الصين، كما تحدث عدة آلاف من حالات سرطانة الأوعية الصفراوية جديدة سنويا. في 18مايو 2014، قام تلفزيون الصين المركزي ببث حلقة حول عادة تناول الأسماك النيئة وتسببها في الإصابة بداء متفرعات الخصية في برنامج تلفزيوني يسمى "الصحة على طرف اللسان". نحن هنا نقدم محتوى البرنامج بإيجاز ونناقش أهميته في السيطرة على داء متفرعات الخصية في الصين

Translated from English version into Arabic by Mahmoud Sami, through

TRANSLATORS
WITHOUT BORDERS

.

华支睾吸虫病防治：从认识开始

钱门宝

摘要：华支睾吸虫病是由于食源性肝吸虫——华支睾吸虫感染引起的，属于被忽视热带病。中国超过 1000 万人感染华支睾吸虫，每年华支睾吸虫感染所致胆管癌高达几千例。2014 年 5 月 18 日，中国中央电视台播放了一期“舌尖上的安全”节目，该节目报道了食用“鱼生”的习惯及其与华支睾吸虫病的关系。本文简要向读者介绍该节目的内容并探讨其对中国华支睾吸虫病防控的意义。

Translated from English version into Chinese by Men-bao Qian

Contrôler de la Clonorchiase par la vigilance

Men-Bao Qian

Sommaire:

La Clonorchiase, infection liée aux aliments, causée par la douve du foie, aussi appelée *Clonorchis sinensis*, peut être également considérée comme l'une des maladies tropicales négligées. On estime à plus de 10 millions le nombre de personnes infectées par le *C. sinensis* en Chine, et plusieurs milliers de nouveaux cas de cholangiocarcinome sont ensuite détectés annuellement. Le 18 Mai 2014, un épisode sur les habitudes de consommer du poisson cru causant la clonorchiase a été diffusé dans un programme de télévision appelé « La Santé sur le bout de la langue », par la chaine chinoise « Central Television ». Nous présentons ici brièvement le contenu du programme et discutons de sa signification concernant le contrôle de la clonorchiase en Chine.

Translated from English version into French by Ode Laforge, through

Борьба с клонорхозом начинается с информированности

Мен-Бао Цянь

Реферат:

Клонорхоз развивается в результате заражения содержащейся в пище китайской двуусткой *Clonorchis sinensis*. Это заболевание можно считать одной из запущенных тропических болезней. По оценкам в Китае заражено *C. sinensis* свыше 10 миллионов человек, и вследствие этого ежегодно возникают несколько тысяч новых случаев холангиогенного рака. Восемнадцатого мая 2014 года Центральное телевидение Китая в программе «Здоровье на кончике языка» показало сюжет о привычке есть сырую рыбу и о том, как это приводит к заболеванию клонорхозом. Здесь приводится краткое описание программы и обсуждается её значение для борьбы с клонорхозом в Китае.

Translated from English version into Russian by Natalia Potashnik, through

El control de la clonorquiasis comienza con la concienciación

Men-Bao Qian

Resumen:

La clonorquiasis es una enfermedad causada por una infección de fasciola hepática transmitida por los alimentos, conocida con el nombre de *clonorchis sinensis*, que también puede considerarse como una de las enfermedades tropicales más olvidadas. Se cree que más de 10 millones de personas están infectadas por la *clonorchis sinensis* en China y, en consecuencia, se producen miles de nuevos casos de colangiocarcinoma cada año. El pasado 18 de mayo de 2014, un programa sobre hábitos de consumo de pescado crudo y cómo este puede provocar clonorquiasis fue emitido en un programa de la China Central Television (televisión central china) llamado *Health on the tip of tongue*. En este artículo introduciremos brevemente el contenido del programa y trataremos su importancia en el control de la clonorquiasis en China.

Translated from English version into Spanish by Nothomb, through

TRANSLATORS
WITHOUT BORDERS
